# Potential for error when relying on administrative data

**DOI:** 10.1093/bjs/znaf139

**Published:** 2025-07-17

**Authors:** Hila Zelicha, Douglas S Bell, Yijun Chen, Edward H Livingston

**Affiliations:** Department of Surgery, Faculty of Health Sciences, UCLA, Los Angeles, California, USA; Department of Medicine, Division of General Internal Medicine, UCLA, Los Angeles, California, USA; Biomedical Informatics Program of the UCLA Clinical and Translational Science Institute (CTSI), UCLA, Los Angeles, California, USA; Department of Surgery, Faculty of Health Sciences, UCLA, Los Angeles, California, USA; Department of Surgery, Faculty of Health Sciences, UCLA, Los Angeles, California, USA

The widespread use of electronic medical records (EMR) and advanced billing systems has made large databases available for analysing medical care outcomes^1–3^. Most administrative databases, such as the National Inpatient Survey and the Centers for Medicare & Medicaid Services data, rely on billing codes to identify diseases or procedures associated with patient care. However, a fundamental limitation of administrative database research is its reliance on billing codes. For any analyses of these databases, the precision and accuracy of disease identification by billing codes should be verified. It is uncommon for this validation step to be included in research reports relying on administrative data.

To assess the accuracy of diagnostic coding as reflecting the true presence of disease, we analysed the EMRs of a large, integrated health system, extracting records from primary care encounters with a primary diagnosis of hernia, with a subset of patients who had abdominal imaging. We focused on abdominal hernia, a common condition that can be reliably confirmed through imaging, making it an ideal model for assessing misclassification. We hypothesized that reliance on billing records in administrative data frequently misidentifies the presence of actual disease.

Patient-level data were extracted from the UCLA Epic Clarity EHR database, excluding restricted individuals (for example celebrities) under UCLA IRB approval (IRB#23-000174). The analytic sample included primary care clinic visits of patients >16 years diagnosed with a hernia between 1 January 2018 and 6 June 2023. Abdominal hernias were identified by ICD-10 codes K40–K46 as the primary diagnosis. Subtypes included diaphragmatic (K44.9), ventral (K42–K43, K45–K46), and inguinal/femoral (K40–K41). When there were multiple hernia diagnoses, the diagnosis listed first was used.

For patients with abdominal imaging (CT/US), hernia diagnoses were confirmed by natural language processing (NLP) of dictated radiology reports. Sentences were parsed, lowercased, and searched for hernia-related terms. Words used to identify hernia from radiology reports: diaphragmatic—hiatal hernia, diaphragmatic hernia, hiatus, Bochdalek hernia, and paraesophageal hernia; ventral—umbilical hernia, umbilicus hernia, ventral hernia, abdominal wall hernia, Spigelian hernia, Richter hernia, quadrant hernia, lateral abdominal hernia, small fat containing upper abdominal hernia, epigastric hernia, and incisional hernia; inguinal—inguinal, groin, and femoral. Presence was determined by keyword context (for example ‘was present’ *versus* ‘no evidence of’), followed by manual review by two authors (H.Z., E.L.). For multiple imaging records, the earliest date was used as the diagnosis. Analyses were conducted using R Studio v4.3.1.

Of 1 362 440 patients whose records were examined, 41 703 had a hernia diagnosis based on encounter ICD-10 diagnostic coding (*Fig. 1*). Of these patients, 28 555 (68%) had corresponding imaging records available. Based on ICD-10 diagnostic codes for outpatient encounters there were: 12 819 (45%) diaphragmatic hernias, 6979 (24%) ventral hernias, and 8757 (31%) inguinal hernias. Imaging verified the presence of hernia in 10 234 cases, yielding a true positive rate of 368% for the diagnosis. Diaphragmatic hernias were verified in 34% of cases labelled as such in clinic encounters, *versus* 44% for ventral hernias and 32% for inguinal hernias (*Fig. 1*).

**Fig. 1:**
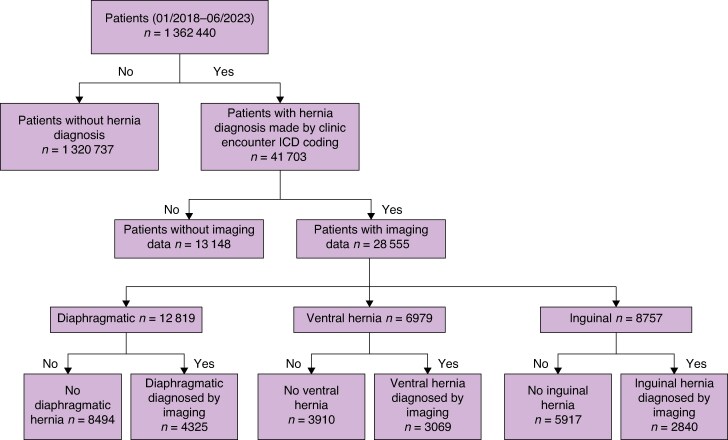
Flow diagram of the clinic encounter ICD coding validated by imaging database

These findings show a very high error rate when assuming a disease entity is present based on ambulatory billing codes found in administrative records. This probably results from clinicians coding visit diagnoses based on a clinical problem being considered, irrespective of its actual presence. For example, if a patient is referred for groin pain because of a possible hernia, the visit will be coded as ‘hernia’ until the diagnosis is ruled out. Although subsequent visits may refine the diagnosis, the erroneous diagnosis remains in the record when retrospectively analysed.

These findings highlight a fundamental weakness in using administrative data for disease identification. Encounter coding occurs because a diagnosis is considered and not necessarily proven. We found that reliance on billing codes for hernia identification could result in two-thirds of cases being erroneously identified. This issue extends beyond hernia^1–5^, highlighting a serious limitation in using administrative data for clinical research. Validation of coding accuracy against actual disease presence is essential before assuming diagnosis validity.

## Data Availability

The de-identified data underlying this article cannot be shared publicly due to the inclusion of personally identifying information.
